# A novel neurodegenerative spectrum disorder in patients with MLKL deficiency

**DOI:** 10.1038/s41419-020-2494-0

**Published:** 2020-05-01

**Authors:** Soren L. Faergeman, Hayley Evans, Kathrine E. Attfield, Christiane Desel, Subita Balaram Kuttikkatte, Mette Sommerlund, Lise Torp Jensen, Jorgen Frokiaer, Manuel A. Friese, Paul M. Matthews, Christian Luchtenborg, Britta Brügger, Annette Bang Oturai, Calliope A. Dendrou, Lars Fugger

**Affiliations:** 1 Oxford Centre for Neuroinflammation, Nuffield Department of Clinical Neurosciences, Division of Clinical Neurology, John Radcliffe Hospital, University of Oxford, Oxford, OX3 9DS UK; 2 MRC Human Immunology Unit, Weatherall Institute of Molecular Medicine, John Radcliffe Hospital, University of Oxford, Oxford, OX3 9DS UK; 3 Department of Clinical Medicine, Aarhus University Hospital, Aarhus, DK-8200 Denmark; 4 Institute of Neuroimmunology and Multiple Sclerosis, University Medical Center Hamburg-Eppendorf, Hamburg, 20246 Germany; 5 Division of Brain Sciences, Department of Medicine, UK Dementia Research Institute, Imperial College London, London, SW7 2AZ UK; 6 Heidelberg University Biochemistry Center (BZH), Heidelberg, D-69120 Germany; 7 Danish Multiple Sclerosis Center, Department of Neurology, Copenhagen University Hospital, Copenhagen, 2100 Denmark; 8 Wellcome Centre for Human Genetics, University of Oxford, Oxford, OX3 7BN UK

**Keywords:** Neurodegeneration, Neurodegenerative diseases

## Abstract

Mixed lineage kinase domain-like (MLKL) is the main executor of necroptosis, an inflammatory form of programmed cell death. Necroptosis is implicated in combating infections, but also in contributing to numerous other clinical conditions, including cardiovascular diseases and neurodegenerative disorders. Inhibition of necroptosis is therefore of therapeutic interest. Here we report two siblings both of whom over the course of 35 years developed a similar progressive, neurodegenerative spectrum disorder characterized by paresis, ataxia and dysarthria. Magnetic resonance imaging of their central nervous system (CNS) revealed severe global cerebral volume loss and atrophy of the cerebellum and brainstem. These brothers are homozygous for a rare haplotype identified by whole genome sequencing carrying a frameshift variant in *MLKL*, as well as an in-frame deletion of one amino acid in the adjacent fatty acid 2-hydroxylase (*FA2H*) gene. Functional studies of patient-derived primary cells demonstrated that the variant in *MLKL* leads to a deficiency of MLKL protein resulting in impairment of necroptosis. Conversely, shotgun lipidomic analysis of the variant in *FA2H* shows no impact on either the abundance or the enzymatic activity of the encoded hydroxylase. To our knowledge, this is the first report of complete necroptosis deficiency in humans. The findings may suggest that impaired necroptosis is a novel mechanism of neurodegeneration, promoting a disorder that shares some clinical features with primary progressive multiple sclerosis (PPMS) and other neurodegenerative diseases. Importantly, the necroptotic deficiency does not cause symptoms outside the nervous system, nor does it confer susceptibility to infections. Given the current interest in pharmacological inhibition of necroptosis by targeting MLKL and its associated pathways, this strategy should be developed with caution, with careful consideration of the possible development of adverse neurological effects.

## Introduction

Mixed lineage kinase domain-like (MLKL) is a pseudokinase that functions as the key effector of necroptosis^1^. Necroptosis is a form of inflammatory programmed cell death that can be triggered by death receptors, toll-like receptors, and viral RNA sensors^2^. Receptor engagement stimulates signal transduction cascades that are regulated by enzymes such as receptor-interacting protein kinase 1 (RIPK1) and RIPK3^3^. Upon activation of RIPK3, MLKL is phosphorylated, allowing it to execute the plasma membrane destabilization that initiates necroptosis^4,5^. Given the molecular pathways that trigger necroptosis, this cell death process may have evolved as a mechanism to combat intracellular pathogens^6–8^. Necroptosis occurs during infection with bacteria, such as *Salmonella enterica*, *Escherichia coli*, *Staphylococcus aureus*, and *Mycobacterium tuberculosis*, and viruses such as human cytomegalovirus can express proteins that actively inhibit necroptotic pathways^8–12^. However, individuals lacking RIPK1 have primary immunodeficiency, lymphopenia, intestinal inflammation, and polyarthritis. These effects have been partly attributed to an increase in necroptosis^13,14^. These reports therefore highlight the relevance of studying patients with genetically determined changes in the necroptosis pathway in order to delineate the mechanisms of the pathway in humans. Indeed, they suggest that dysregulation of necroptosis upstream of MLKL, whether leading to either impairment or enhancement of cell death may be associated with immune abnormalities that alter susceptibility to infection.

Further to their role in the immune system, MLKL activity and necroptosis have also been implicated in the pathogenesis of a range of other clinical conditions, including neurodegenerative diseases such as amyotrophic lateral sclerosis, Alzheimer’s disease, and multiple sclerosis^15–18^. MLKL and other necroptotic pathway proteins are putative therapeutic targets for these and other disorders^2^. Given this perspective, a key question is whether pharmacological inhibition of MLKL and MLKL-dependent pathways could have any adverse effects, particularly as it is emerging that MLKL exerts its function not only by disrupting the plasma membrane integrity during necroptosis, but may also play a role in other cellular processes including vesicle trafficking, inflammasome generation, and cytokine production^19–21^.

In this study we describe two brothers who were originally diagnosed as having primary progressive multiple sclerosis (PPMS), but whose disease development over the course of three decades suggested a novel, progressive neurodegenerative spectrum disorder. In evaluating the possible contribution of genetic factors to the two cases, we found that homozygosity for a haplotype on chromosome 16q23.1 containing two rare, short nucleotide deletions: rs561839347 in the *MLKL* gene and the NM_024306.4:c.32_34del variant in the adjacent *fatty acid 2-hydroxylase* (*FA2H*) gene segregated with disease within the family^22^. Some rare variants in *FA2H* are implicated in leukodystrophy with spastic paraparesis and dystonia, hereditary spastic paraplegia (HSP) form 35, and neurodegeneration with brain iron accumulation (NBIA)^23–27^. We therefore conducted functional studies of the genetic variants in vitro and on patient-derived primary cells. The NM_024306.4:c.32_34del in *FA2H* had no observable impact on FA2H abundance or hydroxylase activity as demonstrated in vitro. In contrast, we found that rs561839347 in *MLKL* was associated with a loss of MLKL protein and complete impairment of necroptosis in our patients. To our knowledge this is the first report of human deficiency of necroptosis being associated with a slowly developing neurodegenerative disease. Furthermore, the patients do not have symptoms from organs outside the nervous system and they do not appear to be prone to infections despite the lack of necroptotic capacity, challenging the concept of MLKL as an important player in the human host response against infectious pathogens^28,29^.

## Materials and methods

### Participants

The two affected brothers developed their first symptoms in the 1980s and they were subsequently diagnosed as having PPMS. As familial PPMS in male patients is rare, the patients were included in a large-scale whole genome sequencing study to identify any rare variants present in both of them that might contribute to their disease^22^. In the current study, these variants were assessed to determine which segregate with disease in the affected family by Sanger sequencing, using the following primers: *FA2H*: TCCCACGTAGTACTGCTCCA and GGTATGCAAATGAGCAGGTG; *MLKL*: CCCTCTAGCCACTGCCAGAAA and CCCTGGCAATTGTGTGTAGCA. Bioinformatic analysis was used to prioritize the variants based on the likelihood of a functional impact and matching with the observed clinical phenotype. The in silico predictive algorithms utilized include PolyPhen-2^30^, phyloP^31^, MutationTaster^32^, and PROVEAN^33^.

The patients underwent neurological examinations in 2013 and 2017. In 2013, magnetic resonance imaging (MRI) scans of their central nervous system (CNS) were also acquired, using a Siemens Avanto 1.5 T scanner, with 3D T1-weighted and T2-weighted imaging, diffusion-weighted imaging and FLAIR imaging. Scans were analysed and formatted using the Horos Dicom Viewer. In 2017, the patients’ full clinical history since the presentation of their first symptoms was evaluated.

This study was approved by the Research Ethical Committee II of Central Denmark Region (Case number 1-10-72-29-16) and the UK NRES Committee South Central—Oxford B (REC Ref. no. 10/H0605/5). Informed consent was obtained from all participants, in accordance with the Declaration of Helsinki; the sister and general medical practitioner of the patients provided consent on their behalf.

### Transfection and lentiviral transduction

Site-directed mutagenesis was used to create constructs carrying the MLKL and FA2H variants according to manufacturers’ protocol (Quickchange II, Agilent). For transient transfection, HEK-293 cells (ATCC) were transfected with hemagglutinin (HA)-tagged MLKL full-length and variant constructs, inserted into the pcDNA3.1+ plasmid (Invitrogen), using Lipofectamine 3000 (Invitrogen). The FA2H variants were inserted into pHRsinUbEm and packaging was performed using a second-generation lentiviral packaging system by co-transfection of packaging plasmid psPAX2 (Addgene plasmid 12260) and vesicular stomatitis virus-G envelop plasmid pMD2.G (Addgene plasmid 12259). CHO-K1 cells (ATCC) were transduced with a multiplicity of infection (MOI) of 20 to express the FA2H variants, and transduction efficiency was >99% for all constructs. Receptor-interacting protein kinase 3 (RIPK3) in pLenti-C-Myc-DDK-P2A-Puro vector (Origene, RC209549L3) was packaged using the same lentiviral packaging system as was used for FA2H. Fibroblasts were transduced with a MOI of 10 to express RIPK3.

### Western blotting

Cell lysates were prepared in RIPA buffer (Sigma-Aldrich) supplemented with cOmplete™, Mini, EDTA-free Protease Inhibitor Cocktail (Roche) according to the manufacturers’ instructions. Blots were stained with monoclonal mouse anti-human FA2H (clone OTI1C5, catalogue number TA506818, Origene), monoclonal rat anti-MLKL (clone 3H1, catalogue number MAC604, Millipore), monoclonal mouse anti-HA (clone 6E2, catalogue number 2367S, Cell Signaling Technology), and monoclonal rabbit anti-human GAPDH (clone 14C10, catalogue number 2118S, Cell Signaling Technology). The secondary antibodies used were anti-mouse IgG-IRDye® 800CW (catalogue number 926-32210) or IgG-IRDye® 680LT (catalogue number 926-68020), anti-rat IgG-IRDye® 800CW (catalogue number 926-32219), and anti-rabbit IgG-IRDye® 680LT (catalogue number 926-68021; all from LI-COR Biosciences).

### Lipidomics

CHO-K1 cells were harvested 72 h after transduction and lipidomic analyses of cell lysates were performed by shotgun lipidomics using the QTRAP6500+system (Sciex) as previously described^34^.

### Primary cell isolation and gene expression analysis

Peripheral blood mononuclear cells (PBMCs) were isolated by density gradient centrifugation. Primary fibroblasts were obtained from skin biopsies taken from the patients and their sister at Aarhus University Hospital, Denmark and were processed in accordance with StemBANCC standard operating procedures (University of Oxford). As an additional control, primary fibroblasts (‘F1’) were purchased from the ATCC (PCS-201-012). Fibroblasts were stimulated with human interferon (IFN)-γ (PeproTech) for MLKL upregulation, and were also cultured with 25 μM of the protease inhibitor MG-132 (Sigma-Aldrich) or 2 μM of the MLKL inhibitor necrosulfonamide (NSA) (Millipore) for 24 h. Real-time quantitative PCR was performed using TaqMan® Gene Expression Assays MLKL_Hs00930421_m1 and GAPDH_Hs02758991_g1. Real-time quantitative PCR was performed using TaqMan® Gene Expression Assays MLKL_Hs00930421_m1 and GAPDH_Hs02758991_g1. Expression of *MLKL* mRNA in PBMCs was assessed in triplicate. Relative transcript levels are expressed as 2^−∆Ct^, where ∆Ct = (MLKL cycle threshold)−(GAPDH cycle threshold). *MLKL* mRNA expression in IFNγ-stimulated fibroblasts was measured in three independent experiments. Protein-level expression was assessed by Western blotting.

### Cell death imaging

Fibroblasts were sensitized to necroptosis by overexpressing RIPK3 by lentiviral transduction and culturing the cells with 5 ng/ml IFNγ (PeproTech) and 50 μM of the pan-caspase inhibitor zVAD (carbobenzoxy-valyl-alanyl-aspartyl-[O-methyl]-fluoromethylketone; Enzo Life Sciences Ltd). Cell death was measured by live-cell imaging using the IncuCyte™ zoom and 250 nM IncuCyte™ Cytotox Red Reagent (Essen Bioscience Ltd).

### Statistical analyses

For the lipidomics two independent analyses were performed, each with three to six independently transduced samples for any single construct. Data were analysed with the ShinyLipids 2015 software (Mathias Gerl, Manuel Haußmann, Sebastian Bender Version 1.0), using a paired, two-tailed *t*-test with a 5% significance threshold. For each FA2H construct tested, we estimated that we would need *n* = 6 to have >95% power to detect a difference between groups of 30% or higher. For cell death imaging analyses a two-way ANOVA Tukey's multiple comparisons test was used with a 5% significance threshold for data analysis, estimating that four experimental replicates would be required to detect an effect size equivalent to a 30% difference between groups with >90% power. Across analyses variance between groups was found to differ and thus assumptions of equal variance were not made. Power calculations were performed with the G*Power software.

## Results

### Clinical characterization reveals a novel neurodegenerative spectrum disorder

The first symptoms of the proband (patient II-3) appeared in 1982 at 19 years of age by asymmetrical weakness in the lower limbs, most notably on his left side. When first admitted to hospital in 1985, he also complained of a transient loss of vision. Clinical examination demonstrated increased muscle tonus of the lower limbs, especially of the left leg, hyperactive reflexes, bilateral ankle clonus, and bilateral Babinski sign. The gait was characterized by spasticity and ataxia. Visual evoked potentials were bilaterally affected with prolonged evoked potentials, whilst sensory evoked potentials were normal. No other sensory deficits were found. The cerebrospinal fluid (CSF) demonstrated a weak oligoclonal band positivity and an increased IgG-index. At the time the patient was diagnosed with PPMS. During the next three decades the patient had a gradual, progressive decline in gait function, eventually requiring permanent use of a wheelchair in 2003. In this time period he also developed paresis, spasticity, and ataxia of the upper limbs. From 1992 he began to have epileptic episodes, in 1995 he developed dysarthria, mild cognitive impairment and urge incontinence. On re-assessment in 2017, the patient had developed fasciculations over the right thigh and severe ataxic dysarthria.

The proband’s older brother (patient II-2) began to experience a disturbance in gait function in 1985 at 30 years of age. Symptoms gradually worsened and in 1990, he had bilateral spasticity of the legs, most prominently on the left side. He had bilateral Babinski sign, but no ataxia or paresis. His arms were unaffected apart from mild hyperreflexia of the left side. Eye examination revealed mild ocular instability and bi-temporally pale discs, but no nystagmus. Given the similarities between his clinical presentation to that of the proband, he was also diagnosed with PPMS, although CSF was not obtained to test for oligoclonal bands. Thereafter, the patient’s ability to walk deteriorated steadily: his maximal walking distance was reduced to 300 m without aids in 1993 and by 1999 he required a wheelchair. During this time period he also developed ataxia of all extremities, foot clonus, urinary incontinence, ataxic dysarthria and mental impairment. All sensory modalities were normal. By 2017, he displayed severe dysarthria, cognitive impairment and marked ataxia and paraparesis of the legs with spasticity (Table 1).

**Table 1:** Patient demographic and clinical characteristics.

	Patient II-3 (Proband)	Patient II-2
Sex	Male	Male
Age of onset (years)	19	30
Disease duration (years)	39	36
Asymmetric weakness of lower limbs	Yes	Yes
Paresis	Yes	Yes
Spasticity	Yes	Yes
Ataxia	Yes	Yes
Sensory symptoms	No	No
Transient loss of vision	Yes	Yes
Ocular instability	Yes	Yes
Bi-temporal pale discs	Yes	Yes
Epilepsy	Yes	No
Dysarthria	Yes	Yes
Mental impairment	Yes	Yes
Urge incontinence	Yes	Yes
Investigations	Isolated oligoclonal band positivity and increased IgG-index (CSF)	–
	Prolonged visual evoked potentials	–
MRI of neuroaxis	Global atrophy	Global atrophy
Symptomatic treatment	Oxcarbazepine	Gabapentin
	Sertraline	Pramipexole
	Tizanidine	Tizanidine
	Tolterodine	

**Fig. 1:**
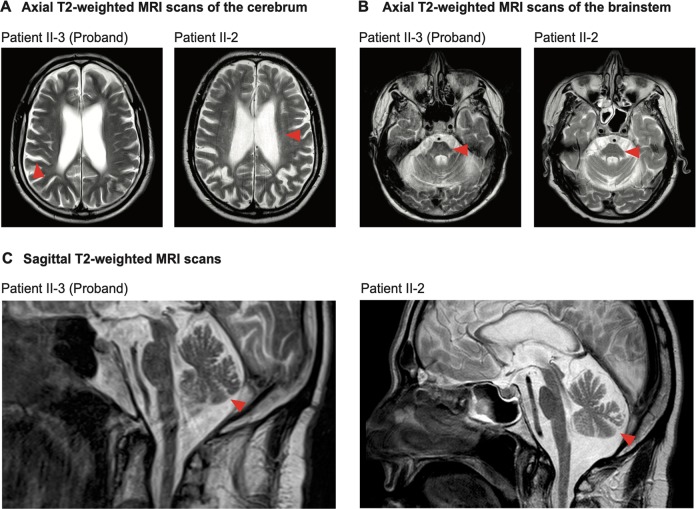
Axial and sagittal T2-weighted MRI scans of the patients’ brains. **a** Axial T2-weighted MRI scans of the patients’ cerebrum. Red arrows denote areas of extensive atrophy. For patient II-2 the red arrow also denotes small, discrete, periventricular T2-hyperintense white matter lesions. **b** Axial T2-weighted MRI scans of the patients’ brainstem. Red arrows denote atrophy in the pons. **c** Sagittal T2-weighted MRI scans of the patients. Red arrows denote atrophy in the cerebellum.

The brothers underwent MRI in 2013 (Fig. 1). The proband’s scan revealed extensive, global atrophy of the cerebrum with dilated sulci, enlargement of the ventricles, atrophy of the corpus callosum and rarefaction of the white matter (Fig. 1a). The cerebellum, the cortical cerebellar fibres, the brainstem, and the middle cerebellar peduncles connecting the cerebellum to the pons were also atrophic (Fig. 1b and c). No regional predominance in atrophy was observed and there were no signs of cerebral ischaemia. There were also no signs of iron deposition in the basal ganglia, which is observed in *FA2H*-associated NBIA. Occasional small, discrete, T2-hyperintense white matter lesions were observed periventricularly. However, these did not have the characteristic morphology or distribution associated with multiple sclerosis as defined in the 2017 McDonald criteria^35^. T2-hyperintense white matter lesions were not observed infratentorially or in the spinal cord. As seen in the scan of the proband, the MRI scan of patient II-2 also showed severe global cerebral, cerebellar, cortical cerebellar fibres tract and brainstem atrophy and small, discrete periventricular T2-hyperintense white matter lesions (Fig. 1).

**Fig. 2:**
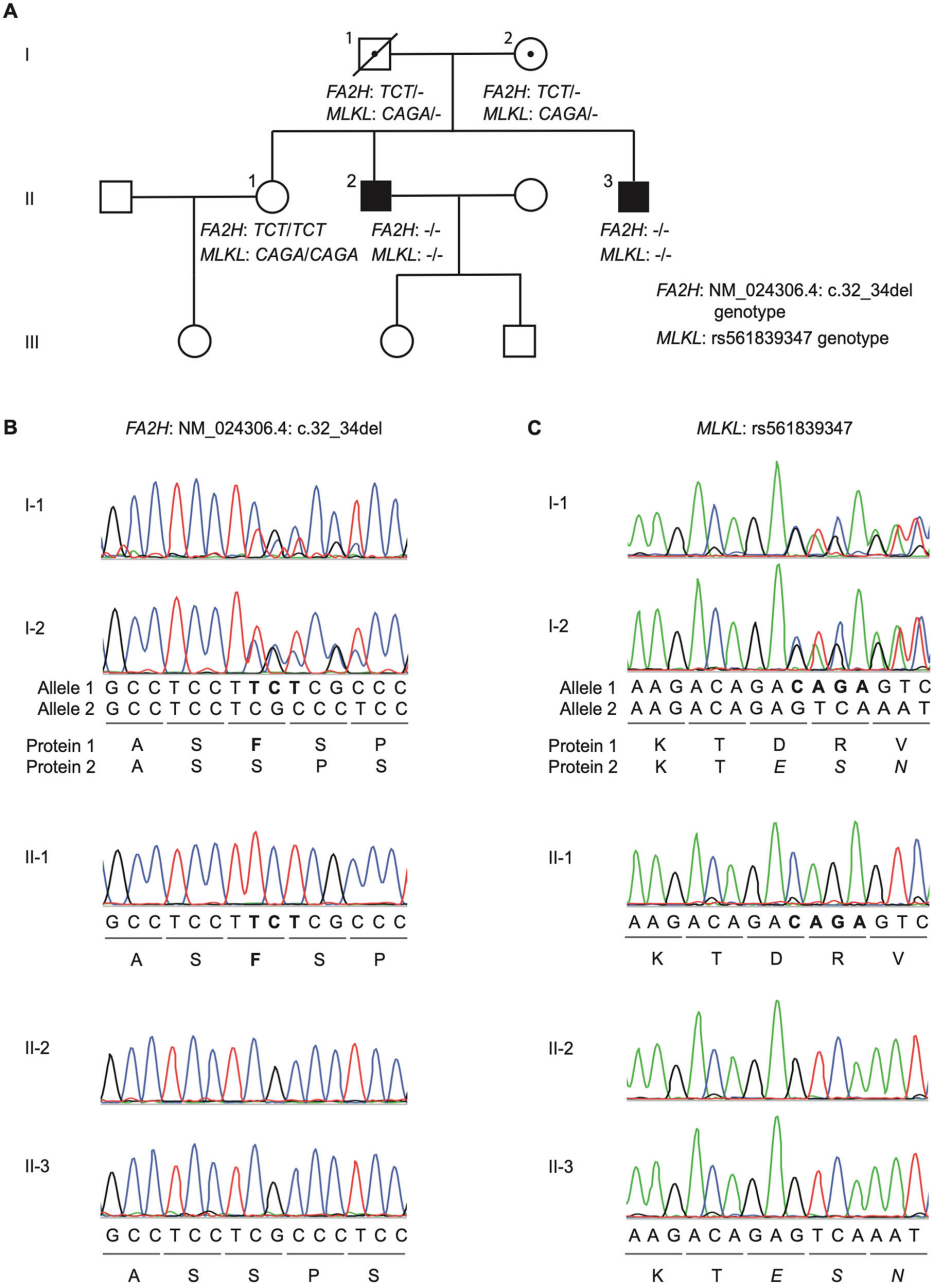
Study family pedigree and *FA2H* and *MLKL* rare variant genotype. **a** Pedigree of the study family showing the segregation of rare, small nucleotide deletions in *FA2H* and *MLKL*. Circles represent women, squares men, the solid symbol represent the clinically affected index patients (brother II-2 and brother II-3, who is the proband), open symbols represent unaffected family members, and dotted symbols indicate heterozygous individuals. A slash through a symbol represents a deceased person; in this family this is the father of the patients, I-1, who passed away in 2015 at the age of 95 years. **b** *FA2H* NM_024306.4:c.32_34del variant genotype of the study family members as determined by Sanger sequencing. Sequences in bold are the three nucleotides and corresponding amino acid that are deleted. **c** *MLKL* rs561839347 variant genotype of the study family members as determined by Sanger sequencing. Sequences in bold represent the four nucleotides that are deleted. Italicized amino acids are novel residues in the predicted protein due to the frameshift.

Collectively, the full clinical history and MRI scans of the two patients are not characteristic of PPMS or of any other specific neurodegenerative spectrum disorder. Nor does the clinical history suggest any primary pathology in organs or tissues outside of the nervous system, any kind of increased susceptibility to infections, or any kind of sensitivity to other environmental factors. No other family members have been diagnosed with a neurodegenerative disease (Fig. 2a). Given the highly similar disease presentation in the two brothers and the absence of an obvious environmental trigger, we hypothesized that rare variants with a recessive homozygous, compound heterozygous or X-linked inheritance pattern could be responsible for their apparently novel neurodegenerative spectrum disorder.

### Genetic analysis identifies rare variants that segregate with disease

The patients had been previously included in a large-scale whole genome sequencing study to identify rare variants present in both of them^16^. Further analysis of these whole genome sequencing data identified rare variants consistent with the hypothesized inheritance patterns, and Sanger sequencing of the unaffected father (I-1), mother (I-2), and sister (II-1) revealed three rare variants that segregated with disease (Fig. 2b and c, and Table S1). The two brothers are homozygous for a chromosome 16q23.1 haplotype carrying the *FA2H* NM_024306.4:c.32_34del and *MLKL* rs561839347 small nucleotide deletions. Their unaffected parents are heterozygous for this haplotype, and their unaffected sister is homozygous for the common haplotype that does not carry these deletions (Fig. 2b and c). The potential functional impact of these variants was assessed bioinformatically using multiple in silico algorithms where possible, and these analyses in conjunction with the described relevance of *FA2H* and *MLKL* to neurodegenerative diseases, suggested a moderate evidence of pathogenicity based on the American College of Medical Genetics and Genomics (ACMG) guidelines^36^. These variants were therefore prioritized for experimental investigation. The third rare variant that segregated with disease is an X-linked, nonsynonymous single nucleotide substitution in *AP1S2*, leading to amino acid substitution K55R. The mother was heterozygous for this variant, whereas it was absent in the father and sister. Bioinformatic analysis provided no strong evidence of a functional effect of this substitution, and as known disease-associated variants in the *AP1S2* gene lead to a distinct, neurodevelopmental rather than neurodegenerative clinical phenotype^37–42^, we considered the variant as likely to be benign based on the ACMG guidelines and the variant was therefore not investigated any further (Table 2).

**Table 2:** Predicted protein level consequences and potential pathogenicity of rare variants in study family.

Predicted change at protein level due to rare variant	Poly Phen-2	phyloP	MutationTaster	PROVEAN	CENTO-GENE	Known clinical phenotypes associated with other variation in gene	Likelihood of pathogenicity based on ACMG guidelines
*MLKL*: Frameshift deletion: 369-471del + novel 21 a/a	N/A	N/A	N/A	N/A	N/A	Heterozygous variant leading to reduced expression associated with late-onset Alzheimer’s disease (see main manuscript for reference)	Moderate evidence of pathogenicity
*FA2H:* Non-frame-shift deletion: F11del	N/A	N/A	N/A	−1.305 (neutral)	Cannot be confirmed as damaging	Multiple *FA2H* variants associated with hereditary spastic paraplegia, leukodystrophy, neurodegeneration with brain iron accumulation (see main manuscript for references)	Moderate evidence of pathogenicity
*AP1S2:* Non-synonymous single nucleotide variant: K55R	0.642 (poss.)	0.99847 (neutral)	0.96041 (neutral)	−2.92 (poss.)	N/A	Variants causing substantial protein truncation or deletion associated with mental retardation, hypotonia, microcephaly, developmental delays with age of onset at 4 years or less	Supporting evidence of benign impact (clinical phenotype mismatch)

The predicted protein level consequences and potential pathogenicity of the rare variants shown in Supplementary Table 1, prior to functional investigations. Potential pathogenicity was assessed using in silico predictive algorithms where possible; these algorithms generate scores for the likelihood of the variant affecting protein function (where ‘poss.’ denotes a possibly damaging effect and ‘neutral’ indicates no likely effect). The *FA2H* non-frameshift deletion F11del was also evaluated by CENTOGENE (www.centogene.com), a CAP accredited company for clinical genetic testing, who could not confirm the variant as damaging. Known clinical phenotypes for other variants in the genes in question are also summarized. The likelihood of variant pathogenicity as estimated by the ACMG guidelines considers variant frequency, potential protein level impact, familial disease pedigree, other known variants in the gene of interest, and the clinical phenotypes they contribute to.

### *FA2H* variant has no impact on hydroxylation of ceramide fatty acids

**Fig. 3:**
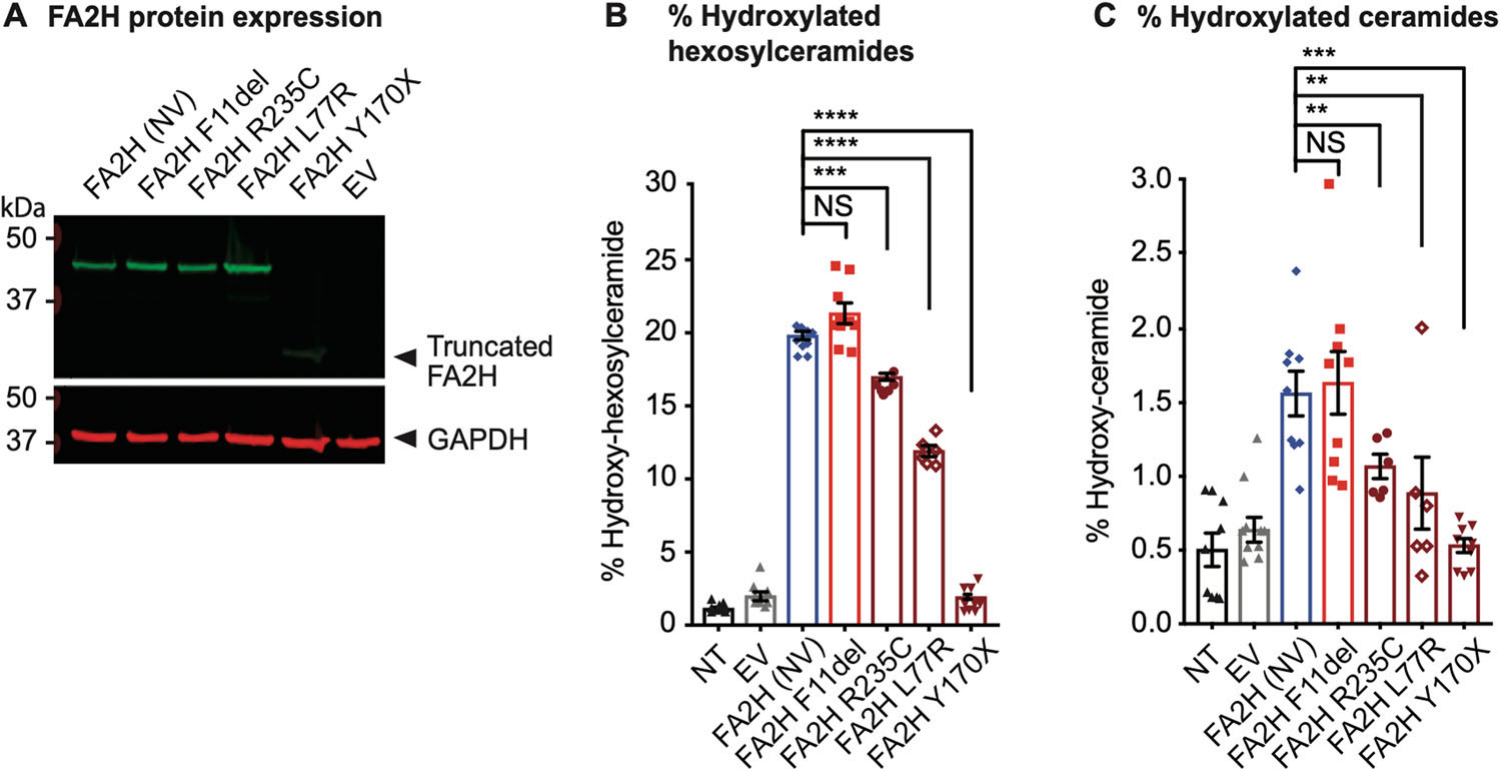
FA2H variant expression and effect on ceramide species hydroxylation. **a** Protein level expression of non-variant (NV) FA2H in relation to the FA2H variant carried by the patients studied here (FA2H F11del), variants described in HSP patients (R235C, L77R and Y170X), and the negative control empty vector (EV) in transduced CHO-K1 cells. The molecular weight, in kilodaltons (kDa), of the truncated low-abundance FA2H Y170X protein, relative to the marker, is denoted by the arrow. GAPDH was used as the loading control. **b** Percentage of hydroxylated hexosylaceramides, relative to the total level of hydroxylated lipids in CHO-K1 cells that were non-transduced (NT), transduced with an empty vector (EV), or transduced to express non-variant (NV) FA2H, the patients’ FA2H protein variant (F11del), or those from hereditary spastic paraplegia patients (R235C, L77R and Y170X). **c** Percentage of hydroxylated ceramides, relative to the total level of hydroxylated lipids in control and transduced CHO-K1 cells. Error bars indicate the standard error of the mean. NS indicates no significant difference. Asterisks indicate a significant difference (**denotes *P* < 0.01; ***denotes *P* < 0.001; ****denotes *P* < 0.0001).

The *FA2H* NM_024306.4:c.32_34del small nucleotide deletion leads to an in-frame deletion of a single amino acid, a phenylalanine at residue 11 (F11del) near the start of the cytochrome b5 haem-binding domain (Fig. 2b and Table 2). The variant has not been previously reported in the Genome Aggregation Database^43^. In conjunction with the transmembrane catalytic region of FA2H, this domain mediates hydroxylation of ceramide fatty acids in the sphingolipid synthesis pathway. To assess the functional impact of the FA2H amino acid deletion found in our patients, CHO-K1 cells were transduced to express the F11del variant, as readily accessible primary cell types do not express FA2H. Results with FA2H F11del were compared with those from the HSP form 35-associated FA2H variants L77R and R235C that are known to reduce enzymatic activity, and to Y170X that leads to a truncated protein lacking the catalytic domain^24–27^. The Y170X truncation was also found to lead to a reduced expression level of this truncated protein, but FA2H F11del showed no altered expression relative to the non-variant protein (Fig. 3a). FA2H enzymatic activity was interrogated by liquid chromatography-tandem mass spectrometry, and unlike the known HSP-associated variants L77R and R235C that are expressed at the same level as F11del, the F11del variant was not associated with a significant reduction in ceramide or hexosylceramide hydroxylation relative to the non-variant protein (Fig. 3b and c). As the F11del variant was not found to impact FA2H expression or enzymatic function, this indicates that the variant is unlikely to be pathogenic.

### *MLKL* variant causes MLKL protein deficiency and impaired necroptosis

The *MLKL* rs561839347 variant is predicted to result in a frameshift, whereby the final 103 residues of the MLKL pseudokinase domain (from amino acid residue 369 onwards) are replaced by a novel 21-amino acid tail (Fig. 2c). The variant has a frequency of 0.42% across all Europeans in the Genome Aggregation Database, which includes three homozygotes of undisclosed clinical phenotype^43^. MLKL constructs lacking the pseudokinase domain have been shown to cause constitutive necroptosis independent of upstream regulators^5,44^, as the pseudokinase domain acts as a negative regulator of MLKL, preventing its function in the absence of an activating signal^3^. Given this, we hypothesized that the predicted truncated MLKL protein would have a gain of function, leading to constitutive necroptosis as a potential mechanism of the accelerated neurodegeneration observed in the patients.

**Fig. 4:**
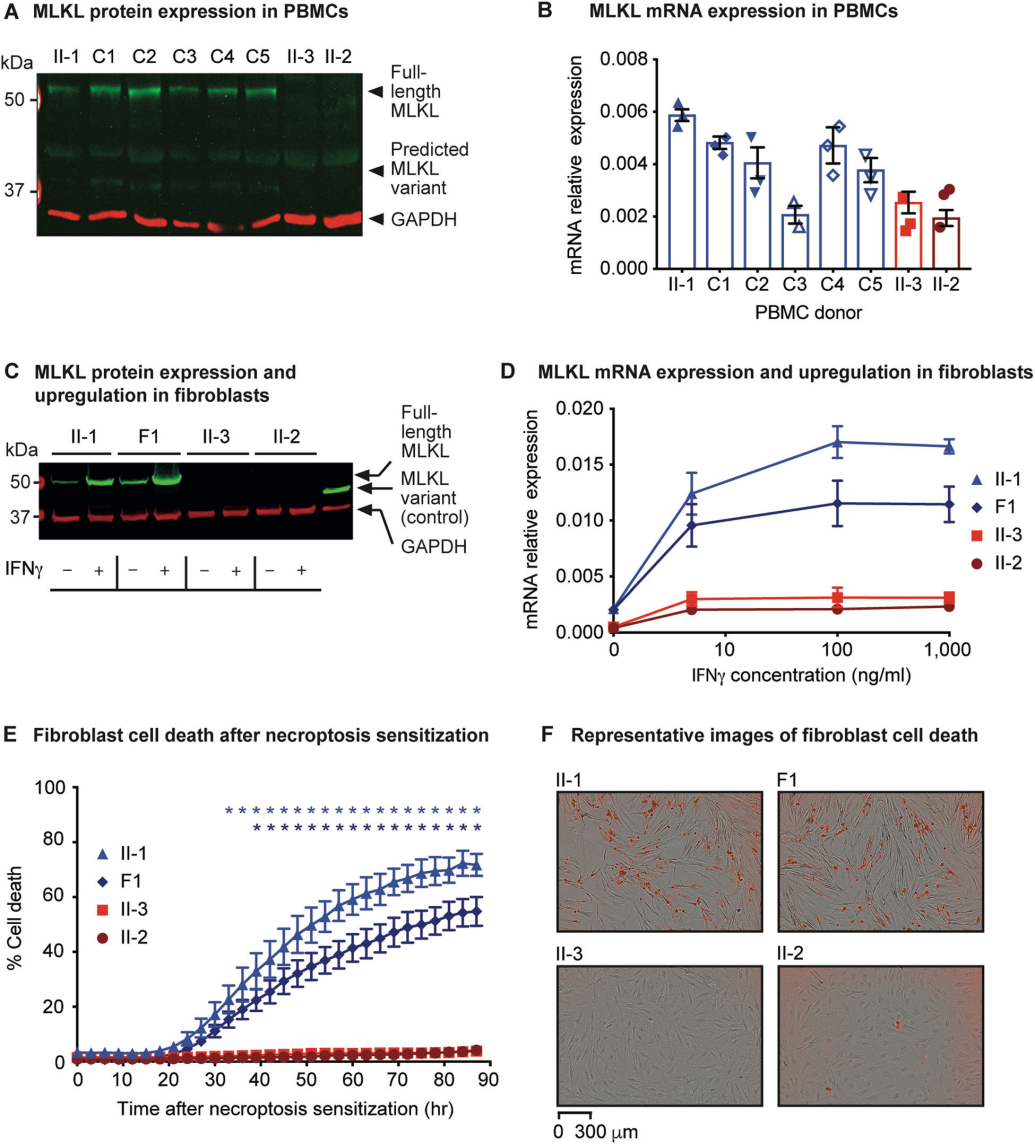
MLKL variant expression and effect on necroptosis. **a** MLKL protein expression in PBMCs of the sister II-1, unrelated controls C1–C5 and the brothers, II-3 and II-2. The molecular weight, in kilodaltons (kDa), of full-length MLKL and of the predicted, truncated MLKL variant (which was not detectable), relative to the marker, are denoted by the arrows. GAPDH was used as the loading control. **b** Relative expression of *MLKL* messenger RNA (mRNA) in PBMCs from controls (sister II-1, and unrelated controls C1–C5; shown in blue) and the patients (brothers II-3 and II-2; shown in red), assessed in triplicate. **c** MLKL protein expression in unstimulated fibroblasts (−) and fibroblasts stimulated with 1000 ng/ml of IFNγ for 24 h (+). Lysate of HEK-293 cells transiently transfected with the truncated MLKL variant protein was used as a control. **d** Relative expression and upregulation of *MLKL* mRNA upon stimulation of fibroblasts from controls (sister II-1 and unrelated control F1; depicted as blue triangles and diamonds, respectively), and the patients (brothers II-3 and II-2; depicted as red squares and circles, respectively) with varying concentrations of IFNγ for 24 h. Three independent experiments were performed. **e** Proportion of dying fibroblasts from the controls (sister II-1 and unrelated control F1; depicted as blue triangles and diamonds, respectively), and the patients (brothers II-3 and II-2; depicted as red squares and circles, respectively) over an 87-h period. Necroptosis sensitization was achieved by RIPK3 transduction in the presence of 5 ng/ml IFNγ and 50 μM zVAD, and cell death was measured based on the uptake of IncuCyte® Cytotox Red Reagent. Four independent experiments were performed. Blue and dark blue asterisks (*) denote a statistically significant difference (*P* < 0.05) between II-I and the patients, and F1 and the patients, respectively. **f** Representative images of the dying fibroblasts (that have taken up the Red Reagent) at the 87-h time point for controls and patients. All images taken are at the same magnification. For all graphs the error bars indicate the standard error of the mean.

To assess the expression of the truncated MLKL protein we used a monoclonal anti-MLKL antibody (from clone 3H1) that binds to the brace region upstream of the MLKL pseudokinase domain^5^ and therefore recognizes both the full-length and the truncated proteins, as validated by an in vitro transfection system (Fig. S1). However, we found that the truncated MLKL protein could not be observed in the PBMCs from the patients, but full-length MLKL was detected in PBMCs from healthy donors, including the patients’ sister, II-1 (Fig. 4a). MLKL mRNA was relatively low in the patients compared to the healthy donors (Fig. 4b), but notably, donor C3 had MLKL mRNA levels that were similar to those of the patients, and yet the MLKL protein was detectable in this individual by Western blotting. This suggests that the absence of MLKL in the patients was unlikely to be due to low MLKL mRNA levels. These findings were not cell type-specific, as no MLKL protein was observed in fibroblasts from the patients either (Fig. 4c). We then reasoned that the inability to detect MLKL protein expression in the patients could be attributed to the truncated MLKL variant driving constitutive necroptosis, such that the absence of MLKL protein expression would only be apparent and would reflect increased necroptosis. We therefore monitored fibroblast cell death in real time by live-cell imaging in the presence of the apoptosis inhibitor zVAD, but we could find no evidence for constitutive cell death of the patients’ cells compared to fibroblasts from their healthy sister II-1 and an unrelated MLKL-expressing primary fibroblast line F1 (Fig. S2).

We next considered whether expression of the truncated MLKL protein could be induced by stimulating the fibroblasts using IFNγ, as this cytokine has previously been shown to both upregulate MLKL and stimulate necroptosis^45–47^. Despite an upregulation of MLKL mRNA upon IFNγ stimulation in the patient and control fibroblasts (Fig. 4d), no MLKL protein was detected in the patients’ cells (Fig. 4c). This was again found not to be related to any constitutive necroptosis: IFNγ stimulation in the presence of zVAD alone or upon additional transduction of RIPK3^8^, which further sensitized the control fibroblasts to cell death, did not promote necroptosis of the patients’ cells (Fig. 4e and f, and Fig. S2). To confirm that the absence of variant MLKL protein was not due to any low-level constitutive necroptosis, IFNγ-stimulated patient and control fibroblasts were incubated with the MLKL inhibitor NSA, and consistent with a lack of necroptosis, no truncated protein could be detected (Fig. S3).

To address how the patients’ small nucleotide deletion in *MLKL* could ultimately lead to a lack of the protein, we incubated IFNγ-stimulated patient and control fibroblasts with the proteasome inhibitor MG-132. No variant MLKL protein could be detected suggesting that the lack of this protein is unlikely to be due to increased protein degradation (Fig. S3). As the *MLKL* rs561839347 variant is located in exon 8 of the gene, which contains a total of 11 exons, and thus is located more than 50–55 nucleotides upstream of the last exon–exon junction, an alternative mechanism for the absence of the variant protein could be nonsense-mediated decay (NMD)^48^. Given that it was possible to detect the truncated MLKL protein in an in vitro transient transfection system that does not recapitulate naturally occurring NMD (Fig. S1), this supports NMD as the potential mechanism by which homozygosity for the *MLKL* rs561839347 variant in the patients leads to loss of MLKL protein thereby preventing necroptosis.

## Discussion

To our knowledge, this is the first study of MLKL deficiency and its effects on necroptosis in humans. We demonstrate that homozygosity for the *MLKL* rs561839347 variant leads to complete lack of MLKL protein and inhibition of necroptosis in vitro. We identify a novel neurodegenerative spectrum disorder associated with *MLKL* rs561839347 homozygosity. Initially the disease presented with symptoms consistent with PPMS. However, the ensuing clinical course and MRI scans are indicative of a distinct disorder, suggesting that the patients’ disease could be considered as a differential diagnosis to PPMS.

As necroptosis is a regulated process that must be triggered by death receptors, toll-like receptors, or viral RNA sensors^2^ and as MLKL necroptotic activity is suppressed when such receptors and sensors are not engaged^3^, MLKL deficiency alone may not result in a pathophysiological phenotype unless it occurs in a context where necroptosis triggers are present. The finding that *Mlkl* knockout mice do not display a spontaneous neurodegenerative phenotype^49^ is consistent with this idea. Thus, we propose that MLKL deficiency promotes the neurodegenerative spectrum disorder in our patients through a context-dependent, modifying effect, contributing to disease development following an initiating trigger. For example, aging, which is difficult to study in mice in ways that are directly relevant to human disease, could provide a disease-promoting context in humans.

This concept may explain some of the conflicting prior evidence regarding the role of necroptosis in neurodegeneration. Genetic and functional studies suggest that variants in the optineurin (*OPTN*) and TANK-binding kinase 1 (*TBK1*) genes, that are associated with amyotrophic lateral sclerosis, may promote axonal degeneration, in part by facilitating the triggering of oligodendrocyte necroptosis, although the relative importance of this compared to other functions of OPTN and TBK1 remains to be fully elucidated^15,50,51^. Conversely, a rare, heterozygous loss-of-function variant in *MLKL* that reduces but does not completely abrogate gene expression has been reported to be associated with late-onset, apolipoprotein E ε4-negative Alzheimer’s disease^52^. The impact of this variant on necroptosis has not been directly investigated, but a reduction in necroptotic cell death would be anticipated, although this would not be expected to be as pronounced as for the patients in our study.

Reduced necroptosis may contribute to neurodegeneration through several mechanisms. For instance, the prolonged survival of pro-inflammatory cells, such as microglial subsets could result in a net neurodegenerative impact, or a reduction in necroptosis could prolong the clearance of damaged cells, enabling the long-term inflammation, and limiting regenerative processes with aging or CNS insults. Such mechanisms may not only be relevant to the neurodegenerative spectrum disorder described in this study and the patients with *MLKL*-associated late-onset Alzheimer’s disease, but also to multiple sclerosis. Tumour necrosis factor (TNF) can induce necroptosis^2^, but risk of multiple sclerosis is associated with a genetically determined reduction in TNF signaling^53^, and TNF antagonistic drugs worsen the disease in clinical trials^54,55^. This suggests that a relative reduction in necroptosis could also contribute to the neurodegeneration observed in multiple sclerosis.

Another hypothesis to explain how MLKL deficiency could promote neurodegeneration is through the loss of a function distinct to necroptosis. It is emerging that MLKL activity may not be restricted to disrupting plasma membrane integrity during necroptosis, but may also include functions in a range of cellular processes including vesicle trafficking, inflammasome generation, and cytokine production^19,20^. Intriguingly, a recent study in mice reports a role for MLKL in promoting nerve regeneration by facilitating myelin breakdown following tissue injury that is unrelated to its action as the mediator of necroptosis^56^. This suggests that loss of MLKL function might prevent regenerative processes after CNS damage. Lastly, an alternative possibility is that although we have found no evidence of rs561839347 homozygosity promoting expression of the truncated MLKL protein in PBMCs and fibroblasts, this protein could nevertheless be weakly expressed in CNS cells and could be constitutively active or could be amenable to alternative modes of activation^57^ thereby leading to low-level, dysregulated necroptosis that could promote CNS pathology.

Further to questioning the role of MLKL in neurodegeneration, our study also has implications for the relative role of necroptosis in combating bacterial and viral infections in humans. The patients have survived into their 6th decade without suffering from recurrent infections, indicating that MLKL and necroptosis in humans may be dispensable for the anti-bacterial and anti-viral capacity of necroptosis. These observations challenge previous studies which, based on human cell lines and *Mlkl* knockout mice, have argued for an important role of necroptosis in the host response against pathogens^7,11^. Notably, our data imply that if MLKL inhibitors were to be used for treating certain patients, one would not anticipate to observe severe immunodeficiency as a result of this treatment, although neurological side effects could arise.

Our approach illustrates how next generation sequencing data analysis combined with functional investigations can be used to delineate a new disease entity. We describe what we believe to be a novel neurodegenerative disease that differs from the original diagnosis of PPMS. With the introduction of the first treatment for PPMS^58^, the accurate distinction of this disease from other neurodegenerative conditions could be important for patient management. More research is necessary to better understand the role of necroptosis across different neurodegenerative diseases: whilst pharmacological inhibition of MLKL and other proteins in the necroptotic cascade is a therapeutic strategy under development for the treatment of neurodegenerative and other diseases^2,18,59–61^, our study raises cautions regarding potential adverse consequences of long-term inhibition of MLKL.

## Supplementary information

Supplementary Figure Legends

Supplementary Table 1

Supplementary Figure 1

Supplementary Figure 2

Supplementary Figure 3

## References

[1] Sun L (2012). Mixed lineage kinase domain-like protein mediates necrosis signaling downstream of RIP3 kinase. Cell.

[2] Linkermann A, Green DR (2014). Necroptosis. N. Engl. J. Med..

[3] Murphy JM (2013). The pseudokinase MLKL mediates necroptosis via a molecular switch mechanism. Immunity.

[4] Cai Z (2014). Plasma membrane translocation of trimerized MLKL protein is required for TNF-induced necroptosis. Nat. Cell Biol..

[5] Hildebrand JM (2014). Activation of the pseudokinase MLKL unleashes the four-helix bundle domain to induce membrane localization and necroptotic cell death. Proc. Natl Acad. Sci. USA.

[6] Wang X (2014). Direct activation of RIP3/MLKL-dependent necrosis by herpes simplex virus 1 (HSV-1) protein ICP6 triggers host antiviral defense. Proc. Natl Acad. Sci. USA.

[7] Guo H (2015). Herpes simplex virus suppresses necroptosis in human cells. Cell Host Microbe.

[8] Omoto S (2015). Suppression of RIP3-dependent necroptosis by human cytomegalovirus. J. Biol. Chem..

[9] Robinson N (2012). Type I interferon induces necroptosis in macrophages during infection with *Salmonella enterica* serovar Typhimurium. Nat. Immunol..

[10] Roca FJ, Ramakrishnan L (2013). TNF dually mediates resistance and susceptibility to mycobacteria via mitochondrial reactive oxygen species. Cell.

[11] Kitur K (2016). Necroptosis promotes *Staphylococcus aureus* clearance by inhibiting excessive inflammatory signaling. Cell Rep..

[12] Pearson JS (2017). EspL is a bacterial cysteine protease effector that cleaves RHIM proteins to block necroptosis and inflammation. Nat. Microbiol..

[13] Cuchet-Lourenco D (2018). Biallelic RIPK1 mutations in humans cause severe immunodeficiency, arthritis, and intestinal inflammation. Science.

[14] Li Y (2019). Human RIPK1 deficiency causes combined immunodeficiency and inflammatory bowel diseases. Proc. Natl Acad. Sci. USA.

[15] Ito Y (2016). RIPK1 mediates axonal degeneration by promoting inflammation and necroptosis in ALS. Science.

[16] Caccamo A (2017). Necroptosis activation in Alzheimer’s disease. Nat. Neurosci..

[17] Ofengeim D (2015). Activation of necroptosis in multiple sclerosis. Cell Rep..

[18] Yuan J, Amin P, Ofengeim D (2019). Necroptosis and RIPK1-mediated neuroinflammation in CNS diseases. Nat. Rev. Neurosci..

[19] Yoon S, Kovalenko A, Bogdanov K, Wallach D (2017). MLKL, the protein that mediates necroptosis, also regulates endosomal trafficking and extracellular vesicle generation. Immunity.

[20] Gong YN (2017). ESCRT-III acts downstream of MLKL to regulate necroptotic cell death and its consequences. Cell.

[21] Conos, S. A. et al. Active MLKL triggers the NLRP3 inflammasome in a cell-intrinsic manner. *Proc. Natl Acad. Sci. USA*10.1073/pnas.1613305114 (2017). 10.1073/pnas.1613305114 PMC5307433 28096356

[22] Taylor JC (2015). Factors influencing success of clinical genome sequencing across a broad spectrum of disorders. Nat. Genet..

[23] Edvardson S (2008). Mutations in the fatty acid 2-hydroxylase gene are associated with leukodystrophy with spastic paraparesis and dystonia. Am. J. Hum. Genet..

[24] Dick KJ (2010). Mutation of FA2H underlies a complicated form of hereditary spastic paraplegia (SPG35). Hum. Mutat..

[25] Donkervoort S (2014). Phenotypic variability of a likely FA2H founder mutation in a family with complicated hereditary spastic paraplegia. Clin. Genet..

[26] Liao X (2015). SPG35 contributes to the second common subtype of AR-HSP in China: frequency analysis and functional characterization of FA2H gene mutations. Clin. Genet..

[27] Kruer MC (2010). Defective FA2H leads to a novel form of neurodegeneration with brain iron accumulation (NBIA). Ann. Neurol..

[28] Lee ACY (2019). H7N9 influenza A virus activation of necroptosis in human monocytes links innate and adaptive immune responses. Cell Death Dis..

[29] Sai K, Parsons C, House JS, Kathariou S, Ninomiya-Tsuji J (2019). Necroptosis mediators RIPK3 and MLKL suppress intracellular Listeria replication independently of host cell killing. J. Cell Biol..

[30] Adzhubei IA (2010). A method and server for predicting damaging missense mutations. Nat. Methods.

[31] Pollard KS, Hubisz MJ, Rosenbloom KR, Siepel A (2010). Detection of nonneutral substitution rates on mammalian phylogenies. Genome Res..

[32] Schwarz JM, Cooper DN, Schuelke M, Seelow D (2014). MutationTaster2: mutation prediction for the deep-sequencing age. Nat. Methods.

[33] Choi Y, Sims GE, Murphy S, Miller JR, Chan AP (2012). Predicting the functional effect of amino acid substitutions and indels. PLoS ONE.

[34] Ozbalci C, Sachsenheimer T, Brugger B (2013). Quantitative analysis of cellular lipids by nano-electrospray ionization mass spectrometry. Methods Mol. Biol..

[35] Thompson AJ (2018). Diagnosis of multiple sclerosis: 2017 revisions of the McDonald criteria. Lancet Neurol..

[36] Richards S (2015). Standards and guidelines for the interpretation of sequence variants: a joint consensus recommendation of the American College of Medical Genetics and Genomics and the Association for Molecular Pathology. Genet. Med..

[37] Ballarati L (2012). Deletion of the AP1S2 gene in a child with psychomotor delay and hypotonia. Eur. J. Med. Genet..

[38] Cacciagli P (2014). AP1S2 is mutated in X-linked Dandy-Walker malformation with intellectual disability, basal ganglia disease and seizures (Pettigrew syndrome). Eur. J. Hum. Genet..

[39] Carpenter NJ, Brown WT, Qu Y, Keenan KL (1999). Regional localization of a nonspecific X-linked mental retardation gene (MRX59) to Xp21.2-p22.2. Am. J. Med. Genet..

[40] Saillour Y (2007). Mutations in the AP1S2 gene encoding the sigma 2 subunit of the adaptor protein 1 complex are associated with syndromic X-linked mental retardation with hydrocephalus and calcifications in basal ganglia. J. Med. Genet..

[41] Tarpey PS (2006). Mutations in the gene encoding the Sigma 2 subunit of the adaptor protein 1 complex, AP1S2, cause X-linked mental retardation. Am. J. Hum. Genet..

[42] Turner G (2003). Syndromic form of X-linked mental retardation with marked hypotonia in early life, severe mental handicap, and difficult adult behavior maps to Xp22. Am. J. Med. Genet. A.

[43] Lek M (2016). Analysis of protein-coding genetic variation in 60,706 humans. Nature.

[44] Quarato G (2016). Sequential engagement of distinct MLKL phosphatidylinositol-binding sites executes necroptosis. Mol. Cell.

[45] Gunther C (2016). The pseudokinase MLKL mediates programmed hepatocellular necrosis independently of RIPK3 during hepatitis. J. Clin. Invest..

[46] Cekay, M. J. et al. Smac mimetics and type II interferon synergistically induce necroptosis in various cancer cell lines. *Cancer Lett*. 10.1016/j.canlet.2017.09.002 (2017). 10.1016/j.canlet.2017.09.002 28923396

[47] Thapa RJ (2013). Interferon-induced RIP1/RIP3-mediated necrosis requires PKR and is licensed by FADD and caspases. Proc. Natl Acad. Sci. USA.

[48] Lykke-Andersen S, Jensen TH (2015). Nonsense-mediated mRNA decay: an intricate machinery that shapes transcriptomes. Nat. Rev. Mol. Cell Biol..

[49] Wu J (2013). Mlkl knockout mice demonstrate the indispensable role of Mlkl in necroptosis. Cell Res..

[50] Markovinovic, A. et al. Optineurin in amyotrophic lateral sclerosis: multifunctional adaptor protein at the crossroads of different neuroprotective mechanisms. *Prog. Neurobiol*. 10.1016/j.pneurobio.2017.04.005 (2017). 10.1016/j.pneurobio.2017.04.005 28456633

[51] Freischmidt A (2015). Haploinsufficiency of TBK1 causes familial ALS and fronto-temporal dementia. Nat. Neurosci..

[52] Wang B (2018). A rare variant in MLKL confers susceptibility to ApoE varepsilon4-negative Alzheimer’s disease in Hong Kong Chinese population. Neurobiol. Aging.

[53] Gregory AP (2012). TNF receptor 1 genetic risk mirrors outcome of anti-TNF therapy in multiple sclerosis. Nature.

[54] van Oosten BW (1996). Increased MRI activity and immune activation in two multiple sclerosis patients treated with the monoclonal anti-tumor necrosis factor antibody cA2. Neurology.

[55] TNF neutralization in MS: results of a randomized, placebo-controlled multicenter study. (1999). The Lenercept Multiple Sclerosis Study Group and The University of British Columbia MS/MRI Analysis Group. Neurology.

[56] Ying Z (2018). Mixed lineage kinase domain-like protein MLKL breaks down myelin following nerve injury. Mol. Cell.

[57] McNamara DE (2019). Direct activation of human MLKL by a select repertoire of inositol phosphate metabolites. Cell Chem. Biol..

[58] Montalban X (2017). Ocrelizumab versus Placebo in primary progressive multiple sclerosis. N. Engl. J. Med..

[59] Yan B (2017). Discovery of a new class of highly potent necroptosis inhibitors targeting the mixed lineage kinase domain-like protein. Chem. Commun..

[60] Karunakaran D (2016). Targeting macrophage necroptosis for therapeutic and diagnostic interventions in atherosclerosis. Sci. Adv..

[61] Coornaert, I. et al. Novel drug discovery strategies for atherosclerosis that target necrosis and necroptosis. *Expert Opin. Drug Discov*, 1–12, 10.1080/17460441.2018.1457644 (2018). 10.1080/17460441.2018.1457644 29598451

