# Supplementary material for: A novel neurodegenerative spectrum disorder in patients with MLKL deficiency
Source: Cell Death Dis. 2020 May 1;11(5):303. doi: 10.1038/s41419-020-2494-0 (PMC7195448; doi:10.1038/s41419-020-2494-0)
Supplement: Supplementary file 1 — Supplementary Figure Legends [file 41419_2020_2494_MOESM1_ESM.docx]

**Table S1. Rare variants segregating with disease in the study family.**

Given the family pedigree and the clinical presentation of the patients, rare variants that were homozygous, compound heterozygous, or X-linked were hypothesized to contribute to the pathology in patients II-2 and II-3 (proband). Based on this, three variants were identified in the patients and their unaffected parents I-1 and I-2 were genotyped for these variants (Taylor *et al*. 2015 *Nat. Genet*.). In the current study the unaffected sister was also genotyped. Genotypes for each variant are shown below the ID for each family member studied. Chrom., chromosome; Ref., reference (common) allele; Alt., alternative (rare) allele. Chromosome positions are denoted relative to the GRCh37d5 human reference sequence and sequences shown are from the forward strand.

**Figure S1. Anti-MLKL antibody validation.**

HEK-293 cells were transfected with **(A)** empty vector, **(B)** a construct for the expression of untagged, full-length MLKL (MLKL FL) and **(C)** and a construct for the expression of HA-tagged full-length MLKL (HA-MLKL FL). **(D)** Western blot of lysates from HEK-293 cells transfected with full-length MLKL with an N-terminal HA tag, or with HA-tagged variant MLKL. Cells were harvested 8 hours post transfection. Anti-HA staining is visualized in red (left panel). Anti-N-terminal MLKL staining with a monoclonal antibody (from clone 3H1) is visualized in green (middle panel). The yellow bands indicate the overlay between the anti-HA and anti-MLKL staining. Protein molecular weight is shown in kilodaltons (kDa).

**Figure S2. Real-time live-cell imaging of fibroblasts under different conditions.**

(**A-F**) The proportion of dying fibroblasts from the controls (sister II-1 and unrelated control F1; depicted as blue triangles and diamonds, respectively), and the patients (brothers II-3 and II-2; depicted as red squares and circles, respectively) over an 87-hour period under control conditions. Fibroblasts were treated with 5 ng/ml IFNγ (**A**), 50 μM zVAD (**B**), 5 ng/ml IFNγ and 50 μM zVAD (**C**), transduced to express RIPK3 (**D**), transduced and treated with 5 ng/ml IFNγ (**E**), and transduced and treated with 50 μM zVAD (**F**). Three to four independent experiments were performed. Blue and dark blue asterisks (*) denote a statistically significant difference (*P*<0.05) between II-I and the patients, and F1 and the patients, respectively. For all graphs the error bars indicate the standard error of the mean.

**Figure S3. The impact of proteasome and MLKL inhibitors on MLKL variant protein expression.**

Primary fibroblasts from the healthy sister II-1 and patient II-2 were cultured in the presence (+) or absence (–) of 1000 ng/ml of IFNγ, 25 μM of the proteasome inhibitor MG-132, and/or 2 μM of the MLKL inhibitor NSA for 24 hours. MLKL protein expression was detected using Western blotting. The molecular weight, in kilodaltons (kDa), of full-length MLKL and of the predicted, truncated MLKL variant (which was not detectable), relative to the marker, are denoted by the arrows. GAPDH was used as the loading control.
