# Supplementary material for: A novel neurodegenerative spectrum disorder in patients with MLKL deficiency
Source: Cell Death Dis. 2020 May 1;11(5):303. doi: 10.1038/s41419-020-2494-0 (PMC7195448; doi:10.1038/s41419-020-2494-0)
Supplement: Supplementary file 2 — Supplementary Table 1 [file 41419_2020_2494_MOESM2_ESM.docx]

| **Chrom.** | **Gene** | **Position** | **Ref.** | **Alt.** | **I-1** | **I-2** | **II-1** | **II-2** | **II-3** |
| --- | --- | --- | --- | --- | --- | --- | --- | --- | --- |
| 16 | *MLKL* | 74,709,590 | *CTCTG* | *C----*  (rs561839347) | *CTCTG/*  *C----* | *CTCTG/*  *C----* | *CTCTG/*  *CTCTG* | *C----/*  *C----* | *C----/*  *C----* |
| 16 | *FA2H* | 74,808,619 | *GAGA* | *G---* | *GAGA/*  *G---* | *GAGA/*  *G---* | *GAGA/*  *GAGA* | *G---/*  *G---* | *G---/*  *G---* |
| X | *AP1S2* | 15,870,484 | *T* | *C* | *T*/- | *T*/*C* | *T*/*T* | *C*/- | *C*/- |
