# Supplementary figures and images for: A novel neurodegenerative spectrum disorder in patients with MLKL deficiency
Source: Cell Death Dis. 2020 May 1;11(5):303. doi: 10.1038/s41419-020-2494-0 (PMC7195448; doi:10.1038/s41419-020-2494-0)

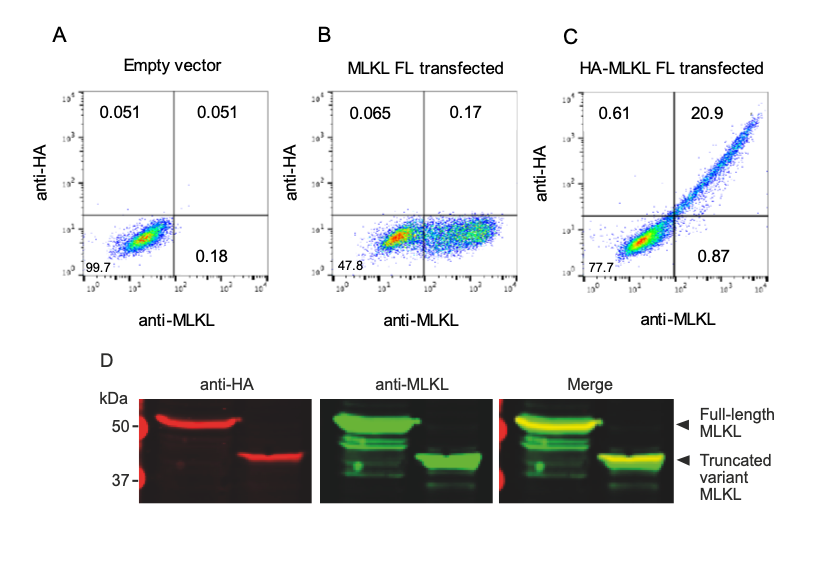

Supplement: Supplementary file 3 — Supplementary Figure 1 [file 41419_2020_2494_MOESM3_ESM.png]

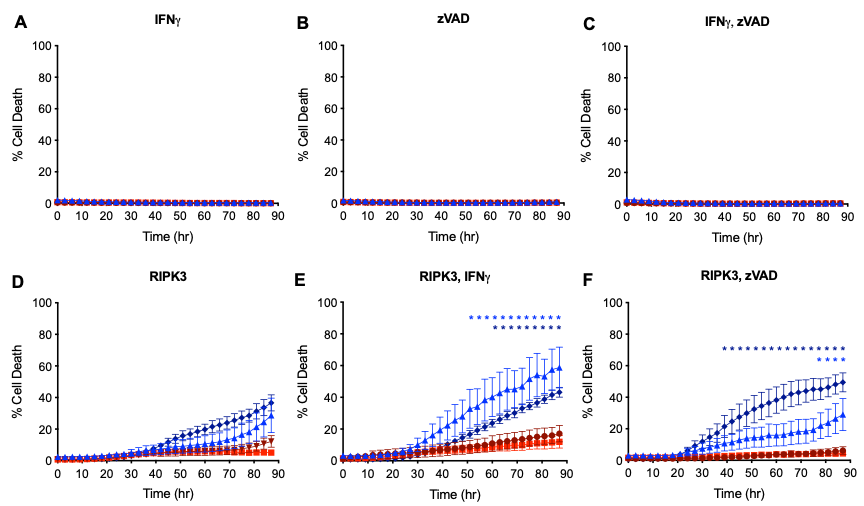

Supplement: Supplementary file 4 — Supplementary Figure 2 [file 41419_2020_2494_MOESM4_ESM.png]

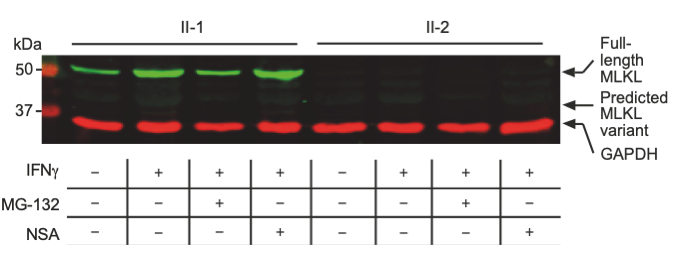

Supplement: Supplementary file 5 — Supplementary Figure 3 [file 41419_2020_2494_MOESM5_ESM.png]
